# Radiation Induced Oral Mucositis

**DOI:** 10.4103/0973-1075.58452

**Published:** 2009

**Authors:** Satheesh Kumar PS, Anita Balan, Arun Sankar, Tinky Bose

**Affiliations:** Department of Oral Medicine and Radiology, Government Dental College, Trivandrum, India; 1 Department of Radiotherapy, Regional Cancer Center, Trivandrum, India

**Keywords:** Mucositis, Oral cancer, Radiation

## Abstract

Patients receiving radiotherapy or chemotherapy will receive some degree of oral mucositis The incidence of oral mucositis was especially high in patients: (i) With primary tumors in the oral cavity, oropharynx, or nasopharynx; (ii) who also received concomitant chemotherapy; (iii) who received a total dose over 5,000 cGy; and (iv) who were treated with altered fractionation radiation schedules. Radiation-induced oral mucositis affects the quality of life of the patients and the family concerned. The present day management of oral mucositis is mostly palliative and or supportive care. The newer guidelines are suggesting Palifermin, which is the first active mucositis drug as well as Amifostine, for radiation protection and cryotherapy. The current management should focus more on palliative measures, such as pain management, nutritional support, and maintenance, of good oral hygiene

## INTRODUCTION

Mucositis is a common complication of cancer therapy, which significantly affects the mucosa. Oral mucositis refers to the oral erythematous and ulcerative lesions commonly observed in patients undergoing cancer therapy. They are painful and affect nutrition and quality of life of the patient, and contribute to local and systemic infections.[1] Radiation-induced changes in the oral and oropharyngeal mucosa have been observed and studied virtually ever since the introduction of radiation as a therapeutic modality.[2] It is often the dose limiting factor, interfering with the intensification of anticancer therapy.[3,4]

Patients for head and neck cancer radiation therapy receive approximately 200 cGy daily dose of radiation, five days per week, for five to seven continuous weeks. Almost all such patients will develop some degree of oral mucositis. Studies show[5,6] severe oral mucositis occurred in 29-66% of all patients receiving radiation therapy for head and neck cancer. The incidence of oral mucositis was especially high in patients: (i) With primary tumors in the oral cavity, oropharynx, or nasopharynx; (ii) who also received concomitant chemotherapy; (iii) who received a total dose over 5,000 cGy; and (iv) who were treated with altered fractionation radiation schedules. The various treatment modalities introduced in order to alleviate the symptoms are enumerated.

## MECHANISM OF DEVELOPMENT

Radiation induced mucositis is initiated by direct injury to basal epithelial cells and cells in the underlying tissue. DNA-strand breaks can result in cell death or injury. Non-DNA injury is initiated through a variety of mechanisms, some of which are mediated by the generation of reactive oxygen species. Radiation and chemotherapy are effective activators of several injury-producing pathways in endothelia, fibroblasts, and epithelia. In these cells, the activation of transcription factors such as nuclear factor-κB (NF-κB) and NRF-2 leads to the upregulation of genes that modulate the damage response. Immune cells (macrophages) produce pro-inflammatory cytokines, such as tumor-necrosis factor-α (TNF-α) and interleukin-6, which causes further tissue injury.[7] These signaling molecules also participate in a positive-feedback loop that amplifies the original effects of radiation and chemotherapy. For example, TNF-α activates NF-κB and sphingomyelinase activity in the mucosa, leading to more cell death. In addition, direct and indirect damages to epithelial stem cells result in a loss of renewal capacity. As a result, the epithelium begins to thin and patients start to experience the early symptoms of mucositis.[8]

An oropharyngeal epithelial surface has a rapid rate of cell turnover and appears to be at high risk of injury from ionizing radiation. A healthy oral mucosa serves to clear microorganism and provides a chemical barrier that limits penetration of many compounds into the epithelium. A damaged mucosal surface increases the risk of a secondary infection. Acute mucositis results from the loss of squamous epithelial cells owing to the sterilization of mucosal stem cells and the inhibition of transit cell proliferation. This leads to a gradual linear decrease in epithelial cell numbers. Normally, cells of the mouth undergo rapid renewal over a 7-14 day cycle. Radiation therapy interferes with cellular mitosis and reduces the ability of the oral mucosa to regenerate.[9]

As radiation therapy continues, a steady state between mucosal cell death and regeneration may occur because of an increased cell production rate from the surviving cells. Usually, however, cell regeneration cannot keep up with cell death, and therefore, partial or complete denudation develops. This presents as patchy or confluent mucositis. As the mucositis becomes more severe, pseudomembranes and ulceration develops. Poor nutritional status further interferes with mucosal regeneration by decreasing cellular migration and renewal. The loss of the epithelial barrier enhances insults from physical, chemical, and microbial agents.

Stages of model[10] for the pathogenesis of mucositis are based on the evidence available to date:

1. Initiation of tissue injury: Radiation and/or chemotherapy induce cellular damage resulting in death of the basal epithelial cells. The generation of reactive oxygen species (free radicals) by radiation or chemotherapy is also believed to exert a role in the initiation of mucosal injury. These small highly reactive molecules are by-products of oxygen metabolism and can cause significant cellular damage.
2. Up-regulation of inflammation via generation of messenger signals: In addition to causing direct cell death, free radicals activate second messengers that transmit signals from receptors on the cellular surface to the inside of cell. This leads to up-regulation of pro-inflammatory cytokines, tissue injury, and cell death.
3. Signaling and amplification: Up-regulation of pro-inflammatory cytokines, such as TNF-α, produced mainly by macrophages, causes injury to mucosal cells, and also activates molecular pathways that amplify mucosal injury.
4. Ulceration and inflammation: There is a significant inflammatory cell infiltrate associated with the mucosal ulcerations, based in part on metabolic by-products of the colonizing oral microf lora. Production of pro-inflammatory cytokines is also further up-regulated as a result of this secondary infection.[10]
5. Healing: This phase is characterized by epithelial proliferation, as well as, cellular and tissue differentiation,[11] restoring the integrity of the epithelium.

A number of authors have reported that the oropharyngeal flora may contribute to radiation-induced mucositis. In health, the oral mucosa has a number of distinct habitats which are colonized by micro-organism that are able to establish a homeostatic community.[12] These homeostatic microbial communities are protective for the host by preventing or interfering with the colonization of exogenous pathogens; this potent defense mechanism is called “colonization resistance”. When the oral tissues are irradiated, the colonization resistance is practically abolished. Irradiation mucositis is caused by a combination of alteration of the normal oral microflora with concomitant changes in the tissues. However, healing eventually occurs when cells regenerate from the surviving mucosal stem cells.

## CLINICAL PRESENTATION

**Figure 1:**
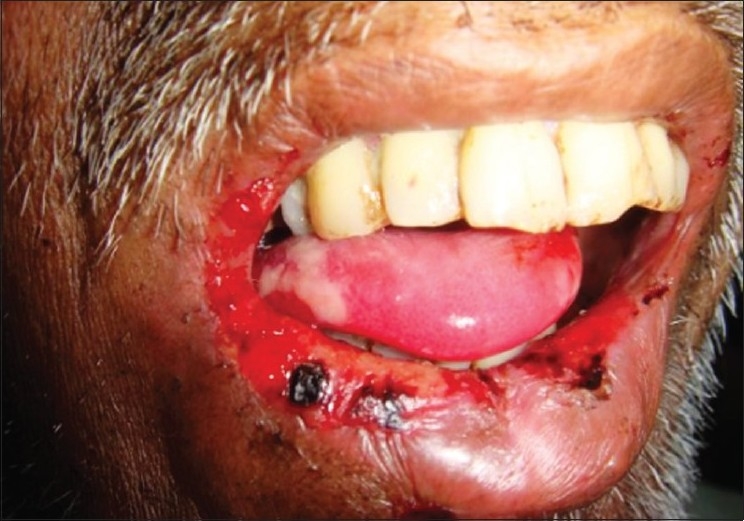
Oral mucositis in a patient who have undergone radiotherapy

Clinically, mucositis presents with multiple complex symptoms. It begins with a symptomatic redness and erythema and progresses through solitary white elevated desquamative patches that are slightly painful to contact pressure. Following this, large, painful contiguous pseudo membraneous lesions develop with associated dysphagia and decreased oral intake. The nonkeratinized mucosa is the most affected one. The most common sites include the labial, buccal, and soft palate mucosa, as well as, the floor of the mouth and the ventral surface of the tongue. Oral lesions usually heal within two to three weeks [Figure 1].

Mucositis is an inevitable side effect of radiation. Its severity is dependent on the type of ionizing radiation, the volume of irradiated tissue, the dose per day, and cumulative dose. It has been noted in a considerable number of clinical trials that the severity of acute normal tissue responses, particularly oral mucositis, is significantly increased when the overall treatment time is shortened.[12,13] The clinical course of oral mucositis may sometimes be complicated by local infection, particularly in immunosuppressed patients. Viral infections such as herpes simplex virus (HSV), and fungal infections such as candidiasis can sometimes be superimposed on oral mucositis. Although HSV infections do not cause oral mucositis, they can complicate its diagnosis and management.

Histopathologically, edema of the retepegs is noted, along with vascular changes that demonstrate a thickening of the tunica intima with concomitant reduction in the lumen size and destruction of the elastic and muscle fibers of the vessel walls. The loss of the epithelial cells to the basement membrane exposes the underlying connective tissue stroma with its associated innervations, which, as the mucosal lesions enlarge, contributes to increasing pain. If the patient develops both severe mucositis and thrombocytopenia, oral bleeding may occur, which is very difficult to treat. Comparison of commonly used mucositis scoring system is possible [Table 1].

**Table 1:** Comparison of commonly used mucositis scoring system

Source	Grade 0	Grade 1	Grade 2	Grade 3	Grade 4
WHO	No change	Soreness/erythema	Erythema, ulcers, can eat solids	Ulcers; requires liquid diet only	Alimentation not possible
RTOG	No change over baseline	May experience mild pain not requiring analgesic	Patchy mucositis may have a serosanguinous discharge. May experience pain requiring analgesics.<1.5 cm, noncontiguous	Confluent fibrinous mucositis/may include severe pain requiring narcotics, > 1.5 cm, contiguous	Necrosis or deep ulceration, ± bleeding
NCI CTC	None	Painless ulcers, erythema or mild soreness	Painful erythema, oedema or ulcers, but can eat	Painful erythema, edema or ulcers cannot eat	Requires parenteral or enteral support
van Der Schueren *et al*.	None	Slight erythema	Pronounced erythema	Spotted mucositis	Confluent mucositis patches >0.5 cm
Byfield *et al*.	-	Minimal dysphasia, thinning but no overt break in mucosal integrity	Significant dysphasia, semi soft foods only, focal mucosal vesicles or denuded patches	Fluids only tolerated, obviously large confluent patches of mucosal denudation	Parenteral fluids only, severe confluent mucosal denudation with bleeding
Seto *et al*.	-	Localized erythema with no pain	Generalized erythema without pain or localized erythema or ulcers with mild pain	Multiple ulcers or generalized erythema with moderate pain	Generalized erwythema or ulcers with moderate to severe pain
Eilers *et al*.	-	Pink and moist	Reddened or white film without ulcerations	Ulceration with or without bleeding	-
NCIC	None	Painless ulcers, erythema, or mild soreness	Painful erythema, oedema, or ulcers, but can eat	Painful erythema, oedema, or ulcers, but cannot eat	Mucosal necrosis and/or requires parenteral or enteral support, dehydration
Spijkervet *et al*.	None	White discoloration	Erythema	Pseudomembrane	Ulceration
Maceijewski	None	Type: Mild erythematous area: <25%	Type: Severe erythematous area: 25-50%	Type: Spotted mucositis area >50%	Type: Confluent mucosistis
Hickey *et al*.	No sto-matitis	Whitish gingival or slight burning sensation or discomfort	Moderate erythema and ulcerations or white patches. Pain, but can eat, drink and swallow	Severe erythema and ulcerations or white patches. Severe pain and cannot eat, drink or swallow	-

### Clinical management of oral mucositis

Management of oral mucositis can be divided into the following sections: Pain control, nutritional support, oral decontamination, palliation of dry mouth, management of oral bleeding, and therapeutic interventions for oral mucositis.

### Pain control

The most common symptom of oral mucositis is pain. Pain significantly affects the nutritional intake, the mouth care, and the quality of life. Thus, management of mucositis pain is a primary component of any mucositis management strategy. Many centers use saline mouth rinses, ice chips, and topical mouth rinses containing an anesthetic, such as 2% viscous lidocaine, which may be mixed with equal volumes of diphenhydramine and a soothing covering agent in equal volumes. Such topical anesthetic agents may provide short-term relief. Sucralfate is the most commonly used and widely studied, even though there is no significant decrease in the pain control.[14,15] In addition to the use of topical agents, most patients with severe mucositis require systemic analgesics, often including opioids, for satisfactory pain relief. Though, the so called ‘*magic mouthwash’* (lidocaine, diphenhydramine, magnesium aluminum hydroxide) has been observed to be beneficial, morphine mouth washes are preferable.[16,17] It was significantly better at reducing intensity and duration of pain and functional impairment, with fewer adverse effects.

## NUTRITIONAL SUPPORT

A soft diet or liquid diet was more easily tolerated than a normal diet, when oral mucositis is present; gastrostomy tube is more beneficial, when there is severe mucositis.

## SELECTIVE ORAL DECONTAMINATION

It has been hypothesized that microbial colonization of oral mucositis lesions exacerbates the severity of oral mucositis and, therefore, decontamination may help to reduce mucositis. Due to the fact that the oral cavity contains a high amount of Gram-negative bacilli and considering its etiological role in mucositis, the concept of ‘Selective Decontamination’ has been developed. In this regard, lozenges composed of polymyxin E, tobramycin, and amphotericin B have been studied in patients receiving radiation for cancers of head and neck in a randomized trial that compared lozenges with placebo or chlorhexidine rinses, the antimicrobial lozenges provided more effective mucositis prevention in patients receiving head and neck irradiation. Addition of ciprofloxacin or ampicillin with clotrimazole to Sucralfate has shown reduction in mucositis.[18]

## ORAL HYGIENE

Significant reduction in oral mucositis can be attained by proper oral hygiene measures.[19] It was noted that proper oral care also reduced oral toxicity of radiation therapy. Indeed, multiple studies have demonstrated that maintenance of good oral hygiene can reduce the severity of oral mucositis. Furthermore, oral decontamination can reduce infection of the oral cavity by opportunistic pathogens.[20] Therefore, a second function of oral decontamination can be to reduce the risk of systemic sepsis from resident oral and/or opportunistic pathogens. Intensive oral care protocol decreased risk of oral mucositis, but not the percentage of patients with a documented septicemia.[21]

The RTOG and MASCC/ISOO (Mucositis study group of the multinational association for supportive care in cancer and the International society of oral oncology) guidelines recommend use of a standardized oral care protocol, including brushing with a soft toothbrush, flossing, and the use of nonmedicated rinses (for example, saline or sodium bicarbonate rinses). Patients and caregivers should be educated regarding the importance of effective oral hygiene.[21]

## PALLIATION OF DRY MOUTH

In cancer therapy, patients often develop transient or permanent xerostomia and hyposalivation. Hyposalivation can further aggravate inflamed tissues, increase risk for local infection, and make mastication difficult. Many patients also complain of a thickening of salivary secretions, because of a decrease in the serous component of saliva. The following measures can be taken for palliation of a dry mouth:

- Sip water as needed to alleviate mouth dryness; several supportive products including artificial saliva are available.
- Rinse with a solution of half a teaspoon of baking soda half in one cup warm water several times a day to clean and lubricate the oral tissues and to buffer the oral environment.
- Chew sugarless gum to stimulate salivary flow.
- Use cholinergic agents as necessary.

## THERAPEUTIC INTERVENTIONS

### Sucralfate

Sucralfate, a cytoprotective agent used for gastro intestinal ulcerations, is a basic aluminum salt of sucrose octasulfate, and may be useful in palliation of established mucositis by its coating and protective actions. This was tried in radio therapy cases by different authors in different combinations with varied results.

### Kaolin pectin

Kaolin pectin, combined with diphenhydramine, which is a H1-histamine antagonist and local anesthetic, was found to reduce oral pain without reducing the degree of mucositis in a double blind randomized and controlled study.[22]

### GROWTH FACTORS

One of the problem faced by the therapy is the loss of proliferation of the oral epithelial cells, it has seen that various growth factors that can increase epithelial cell proliferation have been studied for the management of oral mucositis. Recent evidence shows that intravenous recombinant human keratinocyte growth factor-1, Palifermin, significantly reduced incidence of WHO grades 3 and 4 oral mucositis in patients with hematologic malignancies (for example, lymphoma and multiple myeloma) receiving high-dose chemotherapy and total body irradiation before autologous hematopoietic cell transplantation.[23]

Human keratinocyte growth factor-2, Repifermin, was found to be ineffective in reducing the percentage of subjects who experienced severe mucositis.[24] Intravenous human fibroblast growth factor-20, Velafermin, is currently in clinical development for reduction of mucositis secondary to high-dose chemotherapy in autologous hematopoietic cell transplant patients.[25] The safety of this class of growth factors has not been established in patients with nonhematologic malignancies. There is a theoretical concern that these growth factors may promote growth of tumor cells, which may have receptors for the respective growth factor. However, one recent study found no significant difference in survival between subjects with colorectal cancer receiving Palifermin or placebo at a median follow-up duration of 14.5 months.[26] Further studies are ongoing to confirm the safety of epithelial growth factors in the solid tumor setting, including patients receiving radiation therapy for head and neck cancer.

## ANTI-INFLAMMATORY AGENTS

### Benzydamine hydrochloride

It is a nonsteroidal antiinflammatory drug that inhibits proinflammatory cytokines including TNF-α. In a Phase III trial, Benzydamine hydrochloride mouthrinse reduced the severity of mucositis in patients with head and neck cancer undergoing radiation therapy of cumulative doses up to 50-Gy radiation therapy.[27] Based on this and previous studies, the MASCC/ISOO guidelines recommends use of this agent in patients receiving moderate-dose radiation therapy.[28]

### Saforis

It is a proprietary oral suspension of L-glutamine that enhances the uptake of this amino acid into epithelial cells. Glutamine may reduce mucosal injury by reducing the production of proinflammatory cytokines and cytokine-related apoptosis;[29,30] and may promote healing by increasing fibroblast and collagen synthesis.[31] In a Phase III study, this topical agent reduced the incidence of clinically significant chemotherapy-induced oral mucositis compared to placebo.[32] By comparison, the MASCC/ISOO guidelines recommend that systemically administered glutamine not be used for the prevention of GI mucositis because of lack of efficacy.[33]

### Amifostine

It (phosphothiorate, radiation protection agent) is thought to act as a scavenger for harmful reactive oxygen species that are known to potentiate mucositis.[34] However, because of insufficient evidence of benefit, various guidelines could not be established regarding the use of this agent in oral mucositis in chemotherapy or radiation therapy patients. The use of amifostine has been recommended for the prevention of esophagitis in patients receiving chemoradiation for nonsmall-cell lung cancer.[35]

### RK- 0202

It consists of the antioxidant, *N-acetylcysteine*, in a proprietary matrix for topical application in the oral cavity. In a placebo-controlled phase II trial in patients with head and neck cancer, this agent significantly reduced the incidence of severe oral mucositis up to doses of 50-Gy radiation therapy.[36]

### Beta carotene

Beta carotene, a vitamin A derivative, is a scavenger of singlet oxygen. Based on the findings of different randomized controlled study, it is of the view that supplemental dietary beta-carotene lead to a mild decrease in the severity of chemotherapy and radiotherapy-induced oral mucositis.[37]

## IMMUNOMODULATORY DRUGS

### Pentoxifylline

Oral pentoxiphylline reduced the frequency and severity of all major complications after BMT, including reduction of oral mucositis.[38] Contradictory to this, other workers reported a significant aggravation of symptoms when they studied the effect of IV Pentoxiphylline in 92 patients.[39] However, no difference in symptoms was noted in patients who undergone chemo radio therapy.

### Indomethacin

Indomethacin, a nonsteroidal antiinflammatory drug inhibiting prostaglandin synthesis is noted to delay the onset of mucositis.

### Immunoglobulin

Treatment with low-dose intra muscular immuno globulin is said to decrease the severity and duration of radio therapy-induced oral mucositis. Immunoglobulin has also been tried as a therapeutic agent in radiation- induced mucositis in various clinical trials and the observations were promising.[40]

## CYTOKINES

Preclinical models have been used to demonstrate that the cytokines interleukin-1, interleukin-2, epidermal growth factor, interleukin-11, and transforming growth factor-beta have direct effect on intestinal or oral mucosa. Interleukin-1 increases thymidine labeling, and protects oral and intestinal mucosa, when given to mice before radiation. Interleukin-11 can decrease mucositis, when given to hamster models.

## G-CSF, GM-CSF

The mucosal protection effects of granulocyte colony stimulating factor G-CSF were observed in patients treated with various chemotherapy regimens by many authors.[41] But controversies to this exist in other clinical trials. In a recent preliminary report of a pilot study found significant reduction in oral mucositis.[42] The study was to evaluate the effect of GM-CSF in reduction of radiotherapy induced oral mucositis. At about second week of radiotherapy, when oral pain was experienced 400 μg of GM-CSF was administered locally once a day, until completion of radiotherapy. The patients were evaluated weekly for mucosal reaction and functional impairment. The result of the study was prompting with reduction and almost healing of oral mucositis in 14 out of 17 patients with completion of radiotherapy within the preplanned schedule. Moreover patients did not show a significant weight loss or functional impairment.

## ANTI-VIRAL DRUGS

### Acyclovir

Although acyclovir prophylaxis is effective in preventing oropharyngeal shedding of the virus in herpes simplex virus seropositive patients receiving intensive chemotherapy or BMT, it did not influence chemotherapy, radiotherapy and BMT-related oral toxicity.

## ROLE OF SAFE RADIOTHERAPY

Normal tissue reactions can be reduced in a substantial number of patients with head and neck cancer by the use of computed tomography (CT)-based target delineation, Intensity-Modulated Radiation Therapy (IMRT), and simple, custom-made, intraoral devices that are designed to exclude uninvolved tissues from the treatment portals or to provide shielding of tissues within the treatment area.[43] Stents can be useful in excluding the palate mucosa during treatment of the tongue or floor of the mouth. These shielding stents can decrease the amount of radiation that is delivered to the contra-lateral mucosa. More frequent use of electron-beam and/or sophisticated three-dimensional conformal, multibeam, wedged-pair, or oblique treatment plans will also help to exclude or minimize the radiation dose to uninvolved mucosa. Packing gauze between metallic dental restorations and mucosa of the lateral tongue and buccal area appears to be very beneficial in minimizing the dose from scattered radiation.

## ANTIFUNGAL THERAPY

The mucosa of patients undergoing radiation therapy to the oral cavity should be examined at least once a week, and antibiotic or antifungal medications should be prescribed when infections are documented. Clotrimazole troches, dissolved in the mouth five times a day for 14 days, generally works well for oral candidiasis. However, if significant mucositis, altered taste, or xerostomia has developed, the troches might not be tolerated. In this situation, nystatin oral suspension or Fluconazole in tablet or liquid form is often effective. Fluconazole is more effective than nystatin and might need to be given at a higher dose and/or for an extended period of time in patients who are receiving combined chemotherapy and radiation therapy due to infections with resistant species.[44]

## LOW -LEVEL LASER THERAPY

The mechanism of low-level laser therapy is not understood, but many studies have proved the efficacy of the same in reducing the symptoms related to oral mucositis. Low-level laser therapy may reduce levels of reactive oxygen species and/or proinflammatory cytokines that contribute to the pathogenesis of mucositis.[45] The various guidelines suggest the use of low-level laser therapy for reducing the severity of chemotherapy and radiotherapy-induced oral mucositis.[46]

## NEWER APPROACHES

The new guidelines include Palifermin, the first active mucositis drug, as well as amifostine for radiation protection and cryotherapy for symptoms related to high-dose melphalan.

## CONCLUSION

Radiation-induced oral mucositis affects the quality of life of the patients and the family concerned. The present day management of oral mucositis is mostly palliative and or supportive care. Management includes good oral hygiene, avoiding irritating or abrasive substances, use of bland rinses, topical anesthetic agents, and systemic analgesics. Though, the newer guidelines are suggesting Palifermin, which is the first active mucositis drug as well as Amifostine, for radiation protection and cryotherapy for symptoms related to high-dose melphalan; the role of safe radiotherapy remains the ultimate goal in reducing the symptoms of radiation-induced oral mucositis. Future research for the newer drugs in the field of radiation-induced oral mucositis is a must, and the current management should focus more on palliative measures, such as pain management, nutritional support, and maintenance, of good oral hygiene.
